# Perineural Invasion and Postoperative Adjuvant Chemotherapy Efficacy in Patients With Gastric Cancer

**DOI:** 10.3389/fonc.2020.00530

**Published:** 2020-04-21

**Authors:** Qing Tao, Wen Zhu, Xiaohui Zhao, Mei Li, Yongqian Shu, Deqiang Wang, Xiaoqin Li

**Affiliations:** ^1^Department of Medical Oncology, Affiliated Hospital of Jiangsu University, Zhenjiang, China; ^2^Department of Pathology, Affiliated Hospital of Jiangsu University, Zhenjiang, China; ^3^Department of Medical Oncology, Jiangsu Province Hospital, Nanjing, China

**Keywords:** perineural invasion, gastric cancer, adjuvant chemotherapy, tumor microenvironment, hypoxia, thymidylate synthase

## Abstract

**Purpose:** There is currently a lack of validated predictors for adjuvant chemotherapy efficacy in patients with gastric cancer (GC). Perineural invasion (PNI) is the process of neoplastic invasion of the nerves, accompanied by tumor microenvironment (TME) changes. TME can determine treatment outcome while the impact of PNI on chemotherapy efficacy remains unknown in GC. We investigated the association between PNI and the efficacy of postoperative adjuvant chemotherapy in patients with resected GC.

**Methods:** Patients who underwent radical resection of stage IB-III GC with or without fluoropyrimidine (FU)-based adjuvant chemotherapy were retrospectively selected from two separate patient cohorts. PNI was confirmed with S100 immunohistochemistry (IHC). Tumor hypoxia and activity of selected pathways were quantified by mRNA-based signature scoring based on publicly available data. A hypoxia biomarker, ERO1A, and a FU resistance biomarker, thymidylate synthase (TS), were assessed by IHC, respectively.

**Results:** Two cohorts included 223 and 599 patients, respectively. Adjuvant chemotherapy significantly improved overall survival (OS) and disease-free survival (DFS) in PNI-positive but not in PNI-negative patients, which was not impacted by stages. Multivariate models showed that adjuvant chemotherapy was an independent predictor for OS and DFS in PNI-positive patients in both cohorts. For TME, PNI-negative tumors were more hypoxic than were PNI-positive tumors, and displayed relative up-regulation of signaling along the pathways that are important in FU metabolism or resistance. Expressions of ERO1A and TS significantly decreased in PNI-positive compared to PNI-negative tumors.

**Conclusions:** PNI might help predict adjuvant chemotherapy benefit in patients with resected GC. Validation in prospective studies is required. Novel treatment strategies need to be developed in PNI-negative patients.

## Introduction

China is the world leader in gastric cancer (GC) incidence (1). Currently, radical resection is the main curative method for GC. In East Asia, D2 radical gastrectomy is currently the standard procedure recommended for resectable GC (2). However, surgery alone is insufficient to remove GC and many patients remain at risk of local recurrence and distant metastasis. Randomized phase III clinical trials have demonstrated that fluoropyrimidine (FU)-based adjuvant chemotherapy, following radical gastrectomy, could significantly prolong survival of patients with GC compared to surgery alone (3, 4). Nevertheless, studies have shown that 30–40% of patients treated with surgery and adjuvant chemotherapy relapse within 5 years (3, 4), illustrating variability in response to adjuvant chemotherapy among patients and the need for predictive markers for the efficacy of adjuvant chemotherapy.

Perineural invasion (PNI) is the process of neoplastic invasion of the nerves. PNI is an important marker of local tumor progression, indicative of prognosis in many types of tumors (5, 6). PNI is common in GC; however, the relationship between PNI occurrence and GC prognosis is controversial. A meta-analysis (7) has reported that PNI occurrence affects disease-free survival (DFS) and overall survival (OS) in patients with resected GC. Nevertheless, the strength of evidence upon which this conclusion has been made is weak, as few studies have reported PNI-specific results; in addition, there was high heterogeneity between the studies included in pooled results. In particular, six studies included in this meta-analysis, which involved over 15,000 patients, did not support the independent prognostic role of PNI (7). Of note, the results from these studies were consistent with findings presented in two recent reports (8, 9).

While several studies have investigated the prognostic role of PNI, few studies have investigated the association between PNI and efficacy of adjuvant chemotherapy. In stage II colorectal cancer, Huh (10) found that adjuvant chemotherapy significantly improved 5-year DFS in PNI-positive but not in PNI-negative patients. This finding has been recently confirmed by Cienfuegos et al. (11). Moreover, Suzuki et al. (12) reported similar results in patients with stage III colorectal cancer. Finally, in a recent study, Song et al. (13) revealed that among patients with locally advanced rectal cancer treated with preoperative chemoradiotherapy and radical surgery, PNI-positive patients were more likely than PNI-negative patients to benefit from postoperative adjuvant chemotherapy when distant failure rate was the primary outcome of interest. These results suggest a role of PNI in predicting adjuvant chemotherapy efficacy in colorectal cancer. However, such a role of PNI in GC remains unknown.

The rationality of the potential association between PNI and treatment efficacy is strengthened by progress in understanding molecular mechanisms of PNI. Recent studies emphasize the interactions between perineural microenvironment (PME) and tumor cells (6). PME is composed of neural cells, supporting cells, extracellular matrix, blood vessels, and inflammatory and immune components. Nerve cells and tumor cells can interact directly or through complex signal transduction among various signaling molecules and their receptors. PNI occurs through changes in nerve cells, tumor cells, supporting cells and even the entire PME. Finally, PME evolves into tumor microenvironment (TME). Of note, clinical relevance of TME has been reported in various malignancies (14, 15). Particularly, TME evaluation has been validated as a robust predictor for the efficacy of immunotherapy and adjuvant chemotherapy in GC (16, 17). These findings indicate that PNI may impact treatment efficacy based on TME changes.

In this study, we used two independent patient cohorts to investigate the prognostic and predictive value of PNI in patients with resected GC. We also explored the potential mechanism underlying the clinical significance of PNI, which has not been previously reported, using public RNA sequencing data and a validation protocol for our samples.

## Materials and Methods

### Patients

This study involved two patient cohorts. The Jiangsu Province Hospital (JPH) cohort included 223 patients with GC, diagnosed between 2011 and 2013 at the JPH, Nanjing. The Affiliated Hospital of Jiangsu University (AHJU) cohort included 599 patients with GC, admitted to the AHJU in Zhenjiang city between 2011 and 2016 and screened from a prospective GC database (18).

All patients met the following inclusion criteria: (1) age ≥18 and ≤75 years; (2) history of D2 radical gastrectomy with R0 resection; (3) pathologically-confirmed diagnosis of stage IB-III gastric adenocarcinoma; and (4) FU-based adjuvant chemotherapy or observation during post-surgical follow-up. FU-based adjuvant chemotherapy involved 5-fluorouracil, capecitabine, or S-1 (tegafur-gimeracil-oteracil potassium capsules). Patients were excluded from the study for the following: insufficient clinicopathological data or prior history of radiation therapy, other chemotherapies, or biologic targeted therapies, including neoadjuvant therapy. Clinical and clinicopathologic classification and stage of GC were determined according to the American Joint Committee on Cancer (AJCC) criteria. The hospital's ethics committee approved the research protocol, and all patients signed informed consent.

### PNI Detection

Paraffin-embedded tissue sections of surgical specimens were used to assess PNI, which was defined according to previously reported criteria (5). S100 protein immunohistochemistry (IHC) staining, which is specific for nerves in gastric tissues, was used to assist in the detection of the nerves (19). Hematoxylin and eosin (HE) staining was used to confirm the nerve structure. For each specimen, three independent representative tissue sections were prepared, and the specimens were classified as PNI-positive when PNI was observed in any of the three sections.

### IHC Analysis

Antibodies (Abcam, UK) of S100 (ab52642), thymidylate synthetase (TS, *TYMS*; ab108995) and ERO1A (ab177156) were used for IHC staining with the streptavidin-perosidase method based on a 2-step protocol (20). Positive S100 expression was defined as the presence of nerve staining irrespective of their proportion or intensity, however, S100 staining was generally strong in our samples. Positive TS expression was defined as the presence of nuclear and/or cytoplasm staining of tumor cells irrespective of their proportion or intensity. Positive ERO1A expression had been defined by Seol et al. (21).

### Microarray Data

GC mRNA abundance data from the Asian Cancer Research Group (ACRG) are freely available and were downloaded from the NCBI Gene Expression Omnibus (GEO; GSE62254) and processed as previously described (16). Corresponding PNI data (Table S1) were obtained from the supplementary materials in published literature (22).

### Signature Scoring

Tumor hypoxia were quantified through mRNA-based scoring, using R package known as the *Parametric Gene Set Enrichment Analysis* (PGSEA; http://www.bioconductor.org/), which is based on well-established signatures developed by others, as described elsewhere (23–27). The activities of the selected pathways were also quantified by the same method based on gene sets developed by The Kyoto Encyclopedia of Genes and Genomes (KEGG).

### Statistical Analysis

Overall survival (OS) was defined as the time period from the date of operation to the end of follow-up or cancer-related death. Disease-free survival (DFS) was defined as the time period from the date of operation to the date of recurrence, metastasis, or end of follow-up. A two-sided χ^2^ test, Student's *t*-test, or Mann–Whitney *U*-test, as required, were used to compare the groups of enumeration data. Correlation coefficient was computed by Spearman correlation analysis. Survival analysis was performed using the Kaplan–Meier method plus the Log-rank test. Prognostic factors were determined using univariate and multivariate Cox proportional hazards models. Hazard ratios (HR), along with their 95% confidence intervals (CIs), were calculated. Two-sided *p* < 0.05 was considered statistically significant. We used R (version 3.6.1) and R Bioconductor packages for all analyses.

## Results

### Patient Characteristics

**Figure 1:**
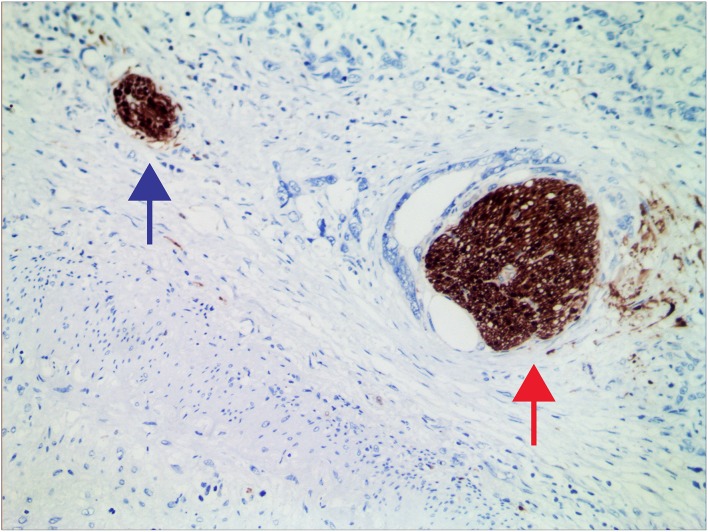
Representative micrographs of nerves invaded (red arrow) and uninvaded (blue arrow) by tumors at 200× magnification.

Typical micrograph of PNI is presented in Figure 1. The incidence of PNI was 33.6 and 56.3% in the JPH and AHJU cohort, respectively (Table 1). In both cohorts, PNI was more frequent in tumors with lymphovascular invasion and TNM stage III (*p* < 0.05). In the AHJU cohort, PNI was also more frequent in cardia tumors and tumors with histologic grade III (*p* < 0.05). Significant heterogeneity was observed for the clinicopathologic characteristics of patients between the JPH and AHJU cohorts, verifying that they were independent.

**Table 1:** Patient characteristics according to status of perineural invasion.

**Characteristic**	**JPH cohort: No (%)**			**AHJU cohort: No (%)**		
	**PNI-**	**PNI+**	***P*-value**	**PNI-**	**PNI+**	***P*-value**
	**148 (66.4)**	**75 (33.6)**		**262 (43.7)**	**337 (56.3)**	
**Age (years)**						
<65	106 (65.4)	56 (34.6)	0.630	159 (46.0)	187 (54.0)	0.201
≥65	42 (68.9)	19 (31.1)		103 (40.7)	150 (59.3)	
**Sex**						
Female	46 (73.0)	17 (27.0)	0.187	74 (48.1)	80 (51.9)	0.211
Male	102 (63.8)	58 (36.3)		188 (42.2)	257 (57.8)	
**Tumor location**						
Non-cardia	96 (66.7)	48 (33.3)	0.898	141 (52.0)	130 (48.0)	<0.001
Cardia	52 (65.8)	27 (34.2)		121 (36.9)	207 (63.1)	
**Histology grade**						
I/II	73 (64.0)	41 (36.0)	0.451	168 (49.7)	170 (50.3)	0.001
III	75 (68.8)	34 (31.2)		94 (36.0)	167 (64.0)	
**Lymphovascular invasion**						
Negative	117 (70.9)	48 (29.1)	0.015	201 (50.1)	200 (49.9)	<0.001
Positive	31 (53.4)	27 (46.6)		61 (30.8)	137 (69.2)	
**TNM stage**						
IB/II	61 (77.2)	18 (22.8)	0.011	151 (63.4)	87 (36.6)	<0.001
III	87 (60.4)	57 (39.6)		111 (30.7)	250 (69.3)	
**Adjuvant chemotherapy**						
Untreated	15 (60.0)	10 (40.0)	0.474	76 (40.0)	114 (60.0)	0.209
Treated	133 (67.2)	65 (32.8)		186 (45.5)	223 (54.5)	

*JPH, Jiangsu Province Hospital; AHJU, Affiliated Hospital of Jiangsu University*.

### Association of PNI With Patient Prognosis

**Figure 2:**
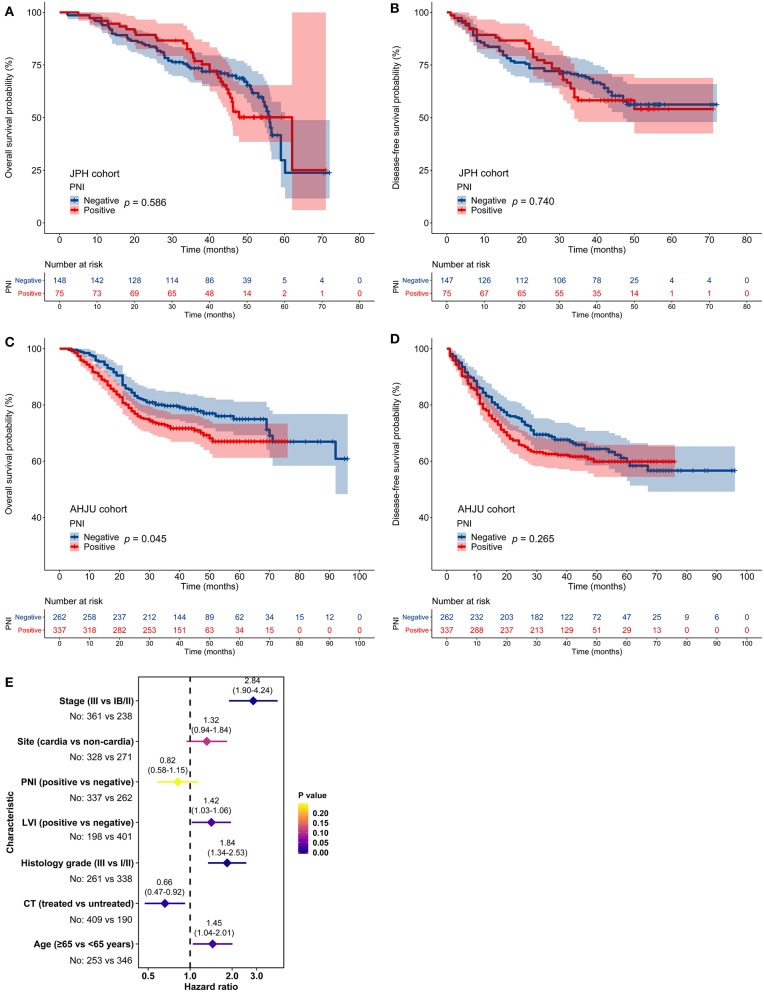
Impact of perineural invasion (PNI) on patient survival. **(A–D)** Overall survival (OS) and disease-free survival in patients with resected gastric cancer according to PNI status. **(E)** Multivariate analyses of variables associated with OS, selected for their prognostic significance established in univariate analysis. LVI, lymphovascular invasion; CT, chemotherapy.

In the JPH cohort, PNI was not associated with OS or DFS (*p* > 0.05; Figures 2A,B). In the AHJU cohort, PNI was associated with poor OS (*p* = 0.045; Figure 2C) but it was not associated with DFS (*p* > 0.05; Figure 2D). However, multivariate analyses showed that PNI was not an independent predictor of OS (HR = 0.82, 95% CI: 0.58–1.15, *p* = 0.243; Figure 2E). Finally, PNI did not correlate with OS and DFS in a combined cohort including all patients (*p* > 0.05; Figure S1).

### Association Between PNI and Chemotherapy Benefit

**Figure 3:**
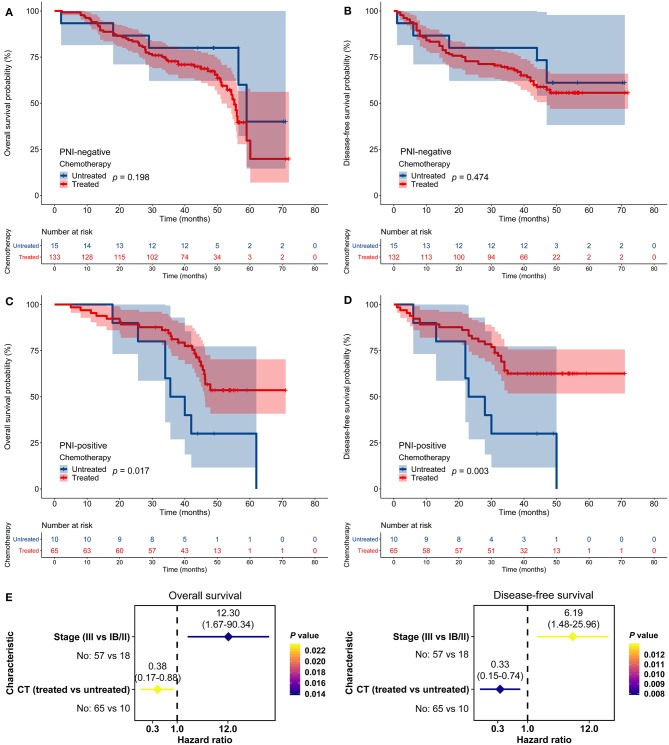
Impact of perineural invasion (PNI) on the efficacy of adjuvant chemotherapy in the Jiangsu Province Hospital cohort. **(A–D)** Overall survival (OS) and disease-free survival (DFS) in patients with resected gastric cancer according to PNI and treatment status. **(E)** Multivariate analyses of variables associated with OS and DFS, selected for their prognostic significance established in univariate analysis. CT, chemotherapy.

In the JPH cohort, FU-based adjuvant chemotherapy did not impact OS or DFS among patients with PNI-negative tumors (*p* > 0.05; Figures 3A,B). In contrast, adjuvant chemotherapy improved OS and DFS among patients with PNI-positive tumors (*p* < 0.05; Figures 3C,D). The multivariate models indicated that adjuvant chemotherapy was an independent predictor for both OS (HR = 0.38, 95% CI: 0.17–0.88, *p* = 0.023) and DFS (HR = 0.33, 95% CI: 0.15–0.74, *p* = 0.008) among patients with PNI-positive tumors (Figure 3E).

**Figure 4:**
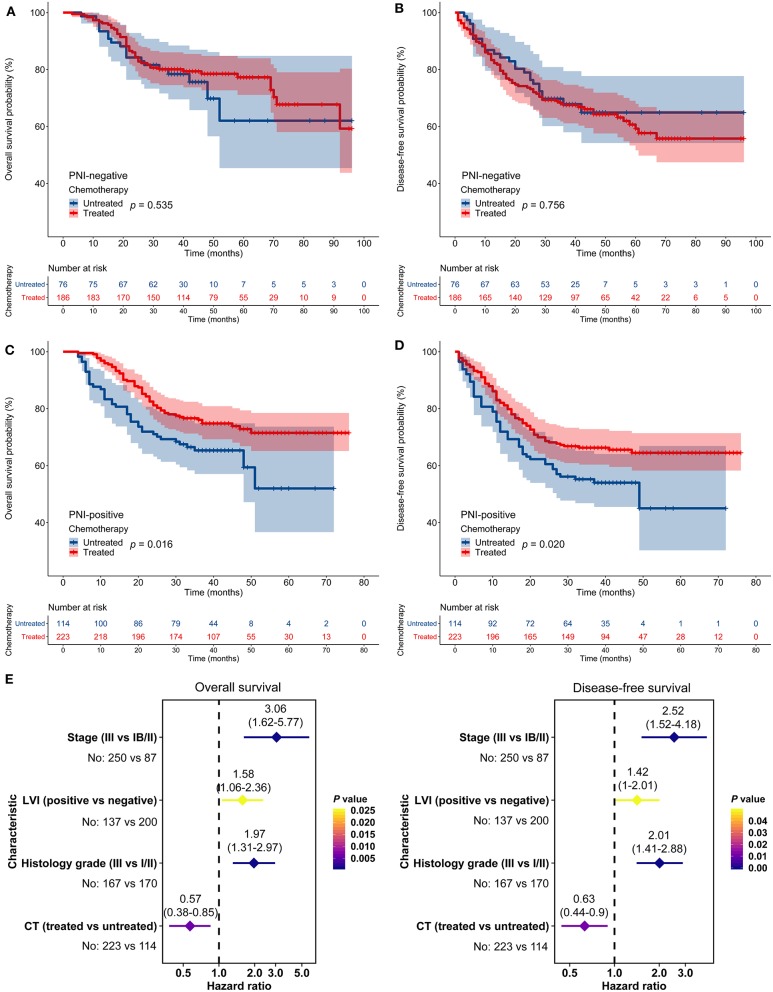
Impact of perineural invasion (PNI) on the efficacy of adjuvant chemotherapy in the Affiliated Hospital of Jiangsu University cohort. **(A–D)** Overall survival (OS) and disease-free survival (DFS) in patients with resected gastric cancer according to PNI and treatment status. **(E)** Multivariate analyses of variables associated with OS and DFS, selected for their prognostic significance established in univariate analysis. LVI, lymphovascular invasion; CT, chemotherapy.

Similarly, in the AHJU cohort, adjuvant chemotherapy improved OS and DFS among patients with PNI-positive tumors but not among patients with PNI-negative tumors (Figures 4A–D). Moreover, adjuvant chemotherapy independently predicted OS (HR = 0.57, 95% CI: 0.38–0.85, *p* = 0.006) and DFS (HR = 0.63, 95% CI: 0.44–0.90, *p* = 0.011) among patients with PNI-positive tumors (Figure 4E). Results for the combined cohort were consistent with findings from separate cohort analyses (Figure S2).

Furthermore, stratified analyses according to stages were conducted in the combined cohort, and we found that patients with PNI-positive tumors benefited from adjuvant chemotherapy in both stage IB/II and stage III while patients with PNI-negative tumors cannot benefit from adjuvant chemotherapy in any stages (Figure S3).

### PNI-Associated TME May Determine Chemotherapy Response

**Figure 5:**
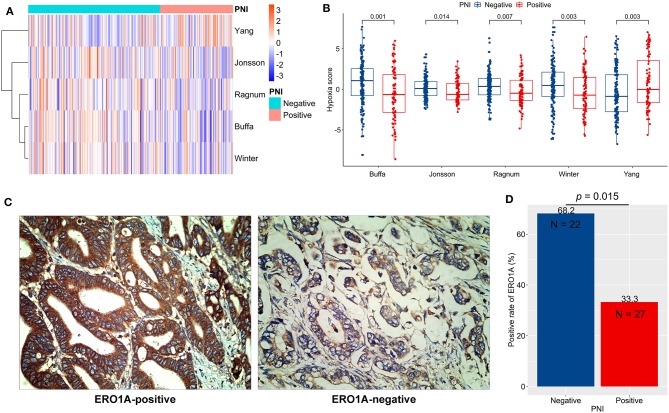
Tumor hypoxia and perineural invasion (PNI). **(A,B)** Differences in hypoxia scores (signature authors are shown) in ACRG between PNI-positive and PNI-negative GC: A clustered heat map **(A)** and direct comparisons **(B)**. **(C)** Typical micrographs of ERO1A-negative and ERO1A-positive tumors, at 400× magnification. **(D)** Different positive rates of ERO1A between PNI-positive and PNI-negative GC. ACRG, Asian Cancer Research Group; GC: gastric cancer.

Hypoxia in TME is a critical determinant of chemotherapy efficacy (28). We quantified tumor hypoxia in ACRG (Table S2), using well-established mRNA-based hypoxia signatures (Figure 5A). PNI-negative tumors were more hypoxic than were PNI-positive tumors (*p* < 0.05; Figure 5B). In contrast, PNI-positive tumors had significantly higher Yang hypoxia signature scores (*p* < 0.05; Figure 5B), which predicted favorable therapy response in patients with bladder cancer (27).

In order to verify the hypoxia difference between PNI-positive and PNI-negative tumors, we detected the protein expression of ERO1A, a novel endogenous marker of hypoxia in cancer (29), in 49 GC tissues of the AHJU cohort (Figure 5C). The ERO1A-positive rate was significantly lower in PNI-positive tumors than in PNI-negative tumors (*p* = 0.015; Figure 5D).

**Figure 6:**
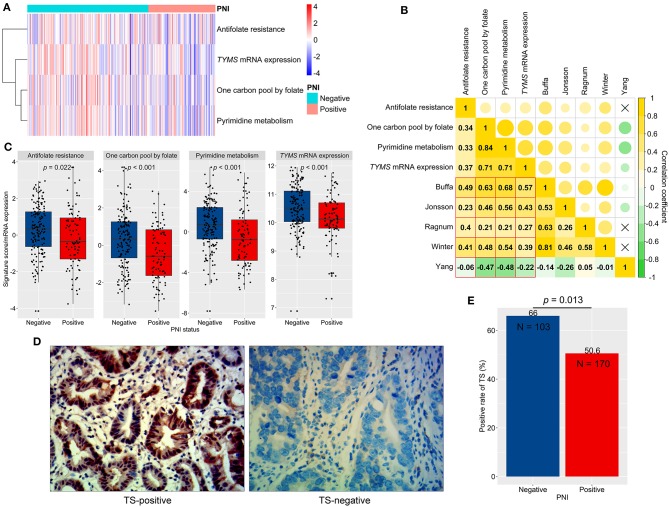
Signaling scores, thymidylate synthetase (TS, *TYMS*) expression, and perineural invasion (PNI). **(A)** A clustered heat map for signaling scores and *TYMS* expression in ACRG. **(B)** The correlations between hypoxia scores (signature authors are shown) and signaling scores (Spearman correlation coefficients were shown and a fork mark indicated non-significant correlation). **(C)** Differences in signaling scores and *TYMS* expression between PNI-positive and PNI-negative GC. **(D)** Typical micrographs of TS-negative and TS-positive tumors, at 400× magnification. **(E)** Different positive rates of TS between PNI-positive and PNI-negative GC. GC, gastric cancer; ACRG, Asian Cancer Research Group.

Hypoxia renders tumors resistant to chemotherapy by affecting signaling crucial for therapy response (28). As a result, we chose to observe the KEGG pathways, which could impact FU metabolism or sensitivity, including entries of pyrimidine metabolism, one carbon pool by folate, and antifolate resistance. We conducted signature scoring for these pathways in ACRG (Figure 6A; Table S3). These pathway scores significantly correlated with hypoxia scores (*p* < 0.05; Figure 6B). Then, we found that PNI-positive tumors obtained significantly lower scores for the pathways of interest than did PNI-negative tumors (*p* < 0.05; Figure 6C).

We next analyzed the relationship between PNI and *TYMS* mRNA expression, a biomarker of FU resistance and a key enzyme in all of the abovementioned pathways (30). We revealed that *TYMS* mRNA significantly correlated with hypoxia scores (Figure 6B) and reduced in PNI-positive compared to PNI-negative tumors (*p* < 0.001; Figure 6C). Furthermore, protein expression of TS, encoding by *TYMS*, was assessed by using IHC in the AHJU cohort (Figure 6D). The TS-positive rate was also significantly lower in PNI-positive tumors than in PNI-negative tumors (*p* = 0.013; Figure 6E).

## Discussion

Previous studies have shown significant genetic and biological heterogeneity among patients with GC, suggesting that prognostic or predictive markers are required to improve patient outcomes. Progress in understanding molecular mechanisms of GC has contributed to the development of novel biomarkers (22, 31), which often involve complex equipment and procedures, as well as time and additional cost. In this study, we showed that an established pathological characteristic, PNI, might help predict the efficacy of adjuvant chemotherapy in patients with resected GC. This finding may help improve patient selection and, consequently, improve therapy outcomes in patients with GC.

Previous studies have reported frequency of PNI occurrence in GC in the range from 6.8 to 75.6% (7), indicating heterogeneity of this patient population as well as variability in accuracy of detection methods, and differences in assessment criteria, among others. In the present study, we used S100 IHC to detect PNI, which is considered a classic approach (19). Moreover, we defined PNI according to Liebig et al's. proposal (5) that incorporated many recognized features of PNI. Adoption of these methods can help decrease the false negative/positive rate in PNI detection.

In our study, both the cardia GC proportion (54.8 vs. 35.4%) and the PNI incidence of this subgroup (63.1 vs. 34.2%) were significantly higher in the AHJU cohort than that in the JPH cohort, which may contribute to the large gap observed for the PNI incidences between these two cohorts. The AHJU cohort and the JPH cohort were heterogeneous, patients in AHJU mainly came from local cities such as Yangzhong city which is well-known for high incidences of gastrointestinal cancers (32), while many patients in JPH came from peripheral provinces and cities because JPH is a regional medical center.

The PME, also known as the perineural niche, is crucial to the pathogenesis of PNI (6, 33). Especially, tumor cells may have passion for PME. First, new tumor cells might prefer to penetrate the soft perineurium rather than the dense desmoplastic microenvironment of the primary tumor. Second, the nutrient-rich perineural space, characterized by extensive vascular and lymphatic supply, might trigger migration of tumor cells growing in a hypoxic and nutrient-deprived microenvironment. These theories suggest that PNI can effectively relieve tumor hypoxia, which is consistent with our finding that PNI-positive tumors were less hypoxic than were PNI-negative tumors.

We further found that in PNI-negative tumors, pathways that are important for FU metabolism or resistance, which could response to hypoxia (34, 35), were upregulated. Finally, in the present study *TYMS* mRNA and TS protein, a target for FU and biomarker of FU resistance (30), were overexpressed in PNI-negative relative to PNI-positive tumors. Recently, in a cohort of 285 GC patients, Pereira (36) found that high TS expression independently predicted poor DFS in stage III GC patients who received 5-FU-based adjuvant chemotherapy. These findings might explain the role of PNI in predicting the efficacy of adjuvant chemotherapy.

Nevertheless, in the present study, PNI was not prognostic in resected GC. Considering the benefits of chemotherapy that were observed among patients with PNI-positive tumors but not among patients with PNI-negative tumors, a potential explanation may be that the adverse impact of PNI on survival could be reversed by adjuvant chemotherapy, which has been reported in patients with colorectal cancer (11, 12). Similarly, the prognostic effect of microsatellite instability (MSI), a potential biomarker for the efficacy of adjuvant chemotherapy in GC, could be attenuated by adjuvant chemotherapy (37). In terms of previous reports about the significant effects of PNI on prognosis (7), we cautioned that some early studies might have been affected by a lack of effective adjuvant therapies among patients with PNI-positive tumors.

This study has some limitations. First, this was a retrospective study, in which patients were not randomly selected. Prospective validation studies are warranted. However, our results were consistent for two independent cohorts, indicating robustness of our findings. Second, PNI might have existed in tumor tissues that were not sampled for testing in this study. Although we used three independent tissue sections to confirm PNI, some false PNI-negative results might have affected our findings. In addition, ethnic differences in the prognostic role of PNI in GC have been reported (7), suggesting the present results need to be verified in non-Chinese populations. Furthermore, data on some clinicopathological features, such as Lauren histotype, were not available in our study.

In conclusion, we revealed that PNI might be an independent predictor for adjuvant chemotherapy efficacy in patients with resected GC; however, its prognostic role remains conflicting. If validated by prospective studies, PNI might become a convenient marker to improve patient selection for adjuvant chemotherapy. Finally, novel treatment strategies need to be developed in PNI-negative patients.

## Data Availability Statement

The data sets in this study that are not in the supplementary material are available from the corresponding author.

## Ethics Statement

The studies involving human participants were reviewed and approved by the ethics committees from Affiliated Hospital of Jiangsu University and Jiangsu Province Hospital. The patients/participants provided their written informed consent to participate in this study.

## Author Contributions

QT, WZ, XZ, ML, YS, DW, and XL were involved in data interpretation and statistical analysis. QT, WZ, DW, and XL were involved in the design of the study and preparation of the manuscript. All authors reviewed and approved the final manuscript.

## Conflict of Interest

The authors declare that the research was conducted in the absence of any commercial or financial relationships that could be construed as a potential conflict of interest.

## Supplementary Material

Figure S1 Impact of perineural invasion (PNI) on patient survival in a combined patient cohort. **(A,B)** Overall survival and disease-free survival among patients with resected gastric cancer according to the PNI status.

Figure S2 Impact of perineural invasion (PNI) on the efficacy of adjuvant chemotherapy in a combined patient cohort. **(A-D)** Overall survival (OS) and disease-free survival (DFS) among patients with resected gastric cancer according to PNI and treatment status. **(E)** Multivariate analyses of variables associated with OS and DFS, selected for their prognostic significance established in univariate analysis. LVI, lymphovascular invasion; CT, chemotherapy.

Figure S3 Impact of perineural invasion (PNI) on the efficacy of adjuvant chemotherapy stratified by stages in a combined patient cohort. **(A–D)** Overall survival and disease-free survival among patients with resected gastric cancer according to PNI and treatment status stratified by stages.

Table S1 Perineural invasion data of gastric cancer samples from the Asian Cancer Research Group.

Table S2 Hypoxia scores of gastric cancer samples from the Asian Cancer Research Group.

Table S3 Selected pathway scores and *TYMS* mRNA expression of gastric cancer samples from the Asian Cancer Research Group.
