# Supplementary figures and images for: Perineural Invasion and Postoperative Adjuvant Chemotherapy Efficacy in Patients With Gastric Cancer
Source: Front Oncol. 2020 Apr 21;10:530. doi: 10.3389/fonc.2020.00530 (PMC7186485; doi:10.3389/fonc.2020.00530)

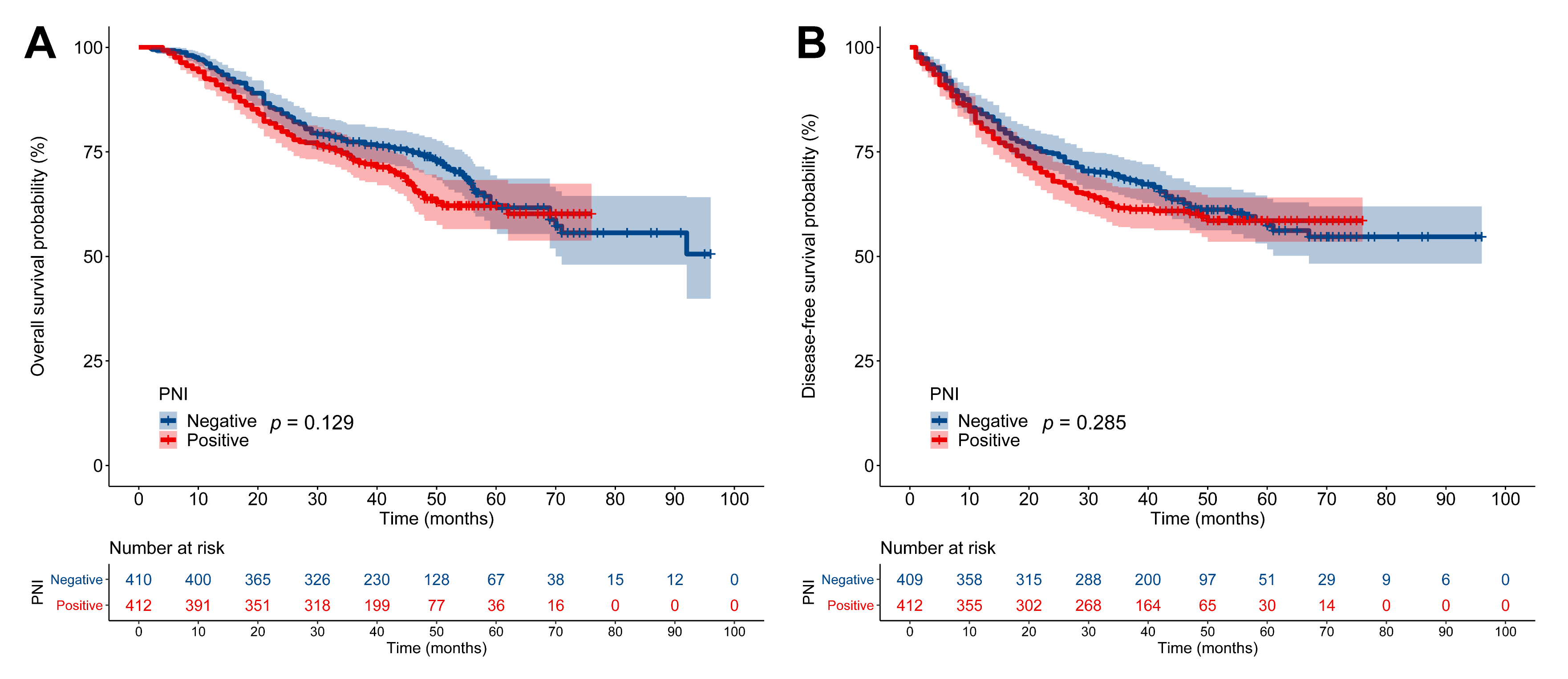

Supplement: Figure S1 — Impact of perineural invasion (PNI) on patient survival in a combined patient cohort. (A,B) Overall survival and disease-free survival among patients with resected gastric cancer according to the PNI status. [file Image_1.tif]

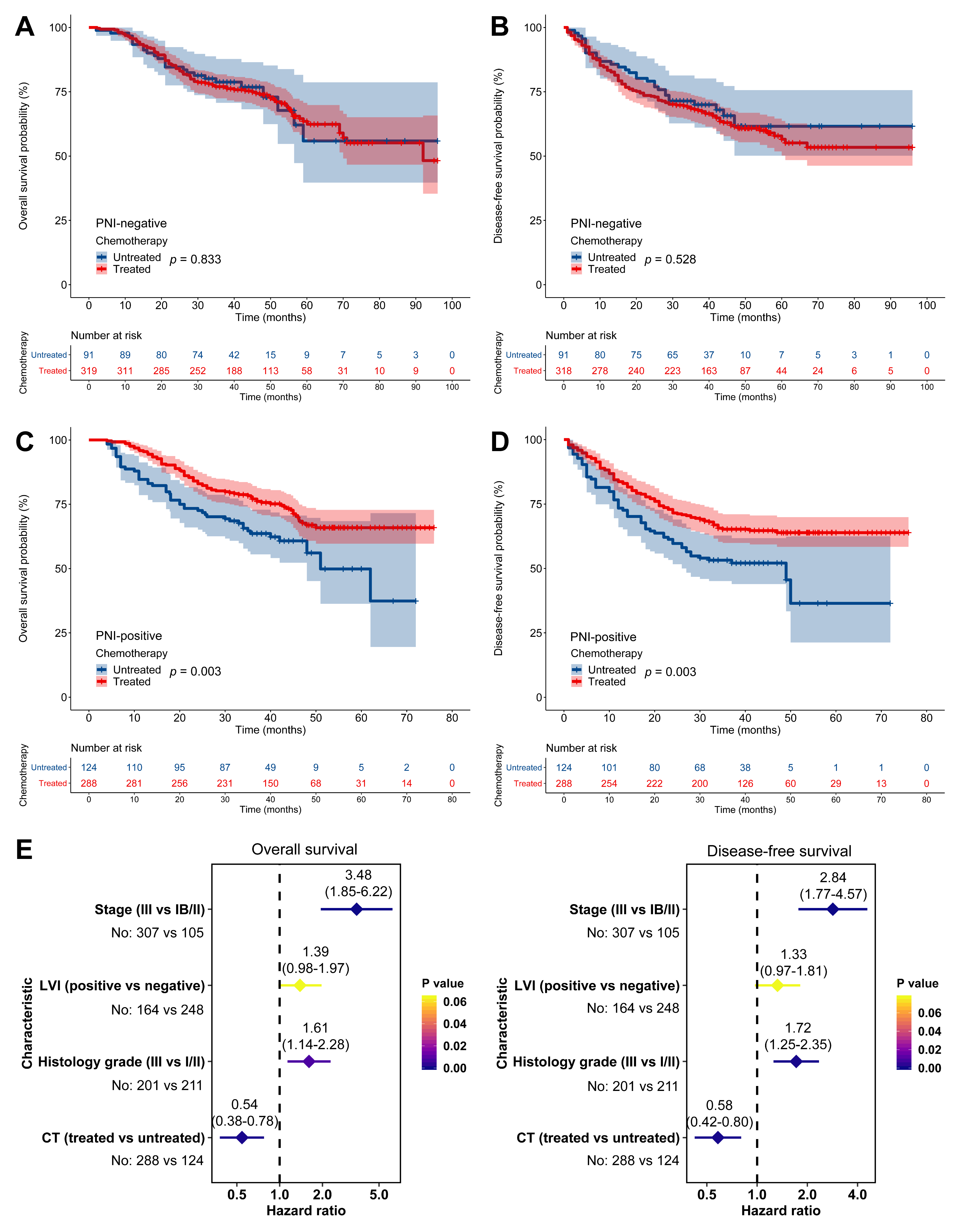

Supplement: Figure S2 — Impact of perineural invasion (PNI) on the efficacy of adjuvant chemotherapy in a combined patient cohort. (A-D) Overall survival (OS) and disease-free survival (DFS) among patients with resected gastric cancer according to PNI and treatment status. (E) Multivariate analyses of variables associated with OS and DFS, selected for their prognostic significance established in univariate analysis. LVI, lymphovascular invasion; CT, chemotherapy. [file Image_2.tif]

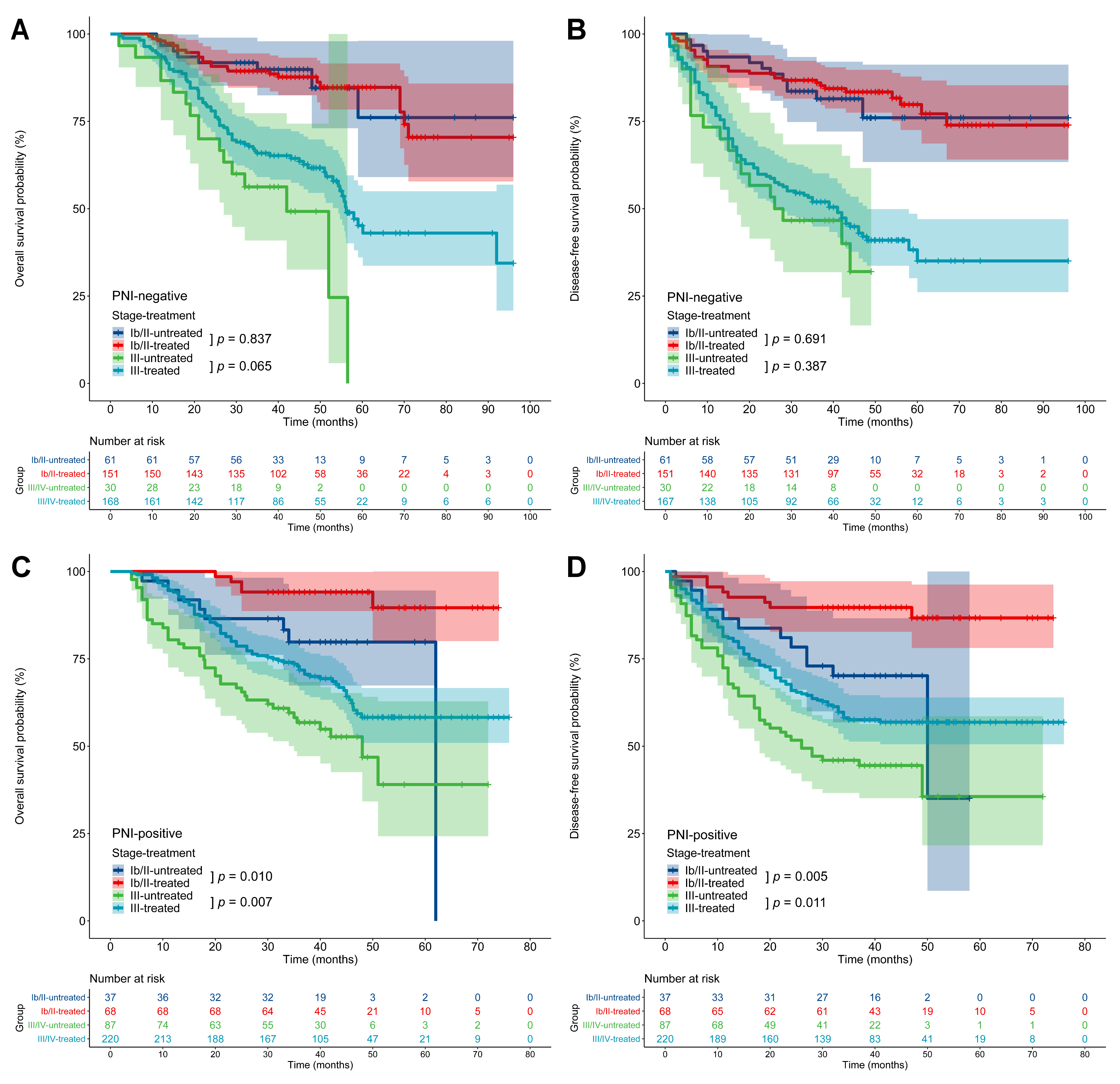

Supplement: Figure S3 — Impact of perineural invasion (PNI) on the efficacy of adjuvant chemotherapy stratified by stages in a combined patient cohort. (A–D) Overall survival and disease-free survival among patients with resected gastric cancer according to PNI and treatment status stratified by stages. [file Image_3.tif]
